# Effect of multiple-compaction on laterite soil: geotechnical and geo-statistical analysis

**DOI:** 10.1038/s41598-024-54644-2

**Published:** 2024-02-19

**Authors:** Imoleayo Oluwatoyin Fatoyinbo, Siyan Malomo, Yinusa Ayodele Asiwaju-Bello, Akinola Akintokunbo Bello

**Affiliations:** 1 College of Civil Engineering, Tongji University, Shanghai, China; 2 Department of Applied Geology, Federal University of Technology, Akure, Nigeria

**Keywords:** Multiple-compaction, Laterite soils, Hardness index, Coefficient of variation, Geology, Civil engineering

## Abstract

Multiple-compaction demonstrates cyclic loading on laterite soils used in highway construction and its effects on engineering properties. The method determines the mechanical stability of derived soils from porphyritic granite, granite gneiss, and charnockite. Fifty-one soil samples were obtained from the horizons of the laterite soil profiles. Four sets of dynamic compactions were carried out on each sample. Index engineering properties such as specific gravity, Atterberg limits, and particle size distribution were investigated before and after multiple-compaction. The changes in engineering properties and moisture-density characteristics were investigated using the granulometric modulus and hardness index. Through multiple-compaction, larger grains in soils derived from granite gneiss and porphyritic granite disintegrated into smaller particles. The fine grains break down more easily than the large grains of quartz bound by clayey materials in charnockite-derived soils. Interestingly, the maximum dry density and optimum moisture content remain consistent in porphyritic and granite gneiss-derived soils after multiple compactions. Based on the densification and behaviour of the derived soils under multiple compaction in highway construction, porphyritic granite and granite gneiss-derived soils are more suitable as engineering materials than charnockite-derived soils.

## Introduction

Laterite soils are soils that have undergone the process of lateralization, resulting in the accumulation of sesquioxides in soil horizons due to weathering. The formation process of laterite is characterized by the physico-chemical breakdown of silicate minerals such as montmorillonite, illite, and kaolinite. The silicate minerals, soluble in alkaline conditions, leach out of the soil profile and the relative proportion of iron or aluminium oxides (sesquioxides) increases significantly in the soil profile. The soils are characterized by a dark brown or reddish-brown due to the presence of iron oxides^1,2^. Laterite soil is unsuitable for construction in its natural state due to its tendency to shrink and expand under varying climate conditions. The soil exhibits different properties depending on the location and evaluation methods used, which can be attributed to the lack of adequate engineering evaluation criteria. Other factors that contribute to the variability of soil include its formation process, degree of weathering, and unique chemical and mineral qualities. It is important to properly evaluate and analyze these factors before considering the use of laterite soil in construction^1,3–5^. The stability and abundance of laterite soil make it a common choice in civil engineering projects within the tropics and subtropics. It's widely available and used in road construction, embankments, earth dams, and airfield pavements^1,6^. Laterite soils consist of unstable coarse grains, which may break down under compaction effort, and silt and clay which are susceptible to swelling and shrinkage. This classification indicates that many laterite soils have anomalous engineering properties that need to be modified^7^**.** The modification of the soil properties through stabilization will improve the strength of the soils^8^. There are two main categories of soil stabilization techniques: mechanical and chemical stabilization. Mechanical stabilization involves modifying soil properties through physical means. The most effective technique in mechanical stabilization is the compaction process. It is imperative to understand that repeatedly compacting and breaking up soil has a profound effect on its properties. Each time the soil is compacted, it becomes more compacted. However, breaking it up before each round of compaction leads to significant changes in its engineering properties from its original state. The removal of the moulding and re-compaction is critical in determining how susceptible the soil is to change under mechanical stress. Over the years, difficulties and variability posed by these soils have been the subject of investigation because of their high vulnerability in causing road failure, and the collapse of bridges, dams, and embankments^1,8–10^. This results in loss of lives and property, and huge costs expended on reconstruction and/or rehabilitation of the failed sections of roads and bridges. The determination of mechanical stability on laterite soils becomes critical due to the presence of coarse and loose-bound micro-cluster grains in the soils that are very sensitive to any form of manipulation such as moulding, wetting, and drying^11^. Previous studies have investigated the effect of compaction on laterite-derived soils. Omotosho and Akinmusuru^6^ studied the behaviour of soils subjected to cyclic compaction. In their study, it was mentioned that cyclic compaction causes a decrease in the liquid limit with each cycle of compaction. With the progression of compaction, the dry density increased, leading to a reduction in both porosity and permeability. Omotosho^12^ investigated the influence of cyclic compaction on the maximum dry density (MDD) and optimum moisture content (OMC) of some deltaic soils used for the construction of road embankments. Samples of these soils were subjected to standard Proctor and break-up Proctor compaction tests. The study proved that standard proctors usually underestimate the Maximum Dry Density (MDD) value, albeit with a decreasing influence as the depth of the sample increases. It was concluded that the recycling procedure involved in most standard laboratory compaction tests tends to underestimate the actual compaction characteristics. He explained that break-up parameters produce greater densities and hence closer approximation to the actual field situations. Ugbe ^11^ investigated the behaviour of soils subjected to compaction. The results of the investigation indicate an increase in the Atterberg limits, maximum dry density, and uniformity coefficient as the compaction cycles increase. Wang et al^13^ investigated the influence of multi-cyclic compaction tests on laterites. They considered the relationship between maximum dry density and optimum moisture content with the number of compactions. They concluded that optimum moisture content does not increase after many compactions while maximum dry density increases when the water content rate reduces after multiple-compaction. Other researchers have employed different compaction energies to examine the effect of compaction on the engineering properties of soils^**,**^^14–17^. The previous research on compaction characteristics is insufficient as it has only focused on coarse-grained soils that are susceptible to degradation during multiple-compaction. This limited approach has resulted in inaccurate estimations, primarily due to the absence of standard compaction assessment techniques. To address this issue, the study conducted a comparative analysis of fine-grained derived soils and coarse-grain derived soils. The study aims to provide a better understanding of the impact of multiple compaction on fine-grained soils, which has been overlooked in previous literature. This study aims to thoroughly analyze the effect of parent rock lithologies on soil engineering properties. It examines how engineering properties differ across various sampling horizons, and evaluates the variations in moisture-density characteristics and grain size distribution of soils from different horizons.

## Geology of the study area

**Figure 1:**
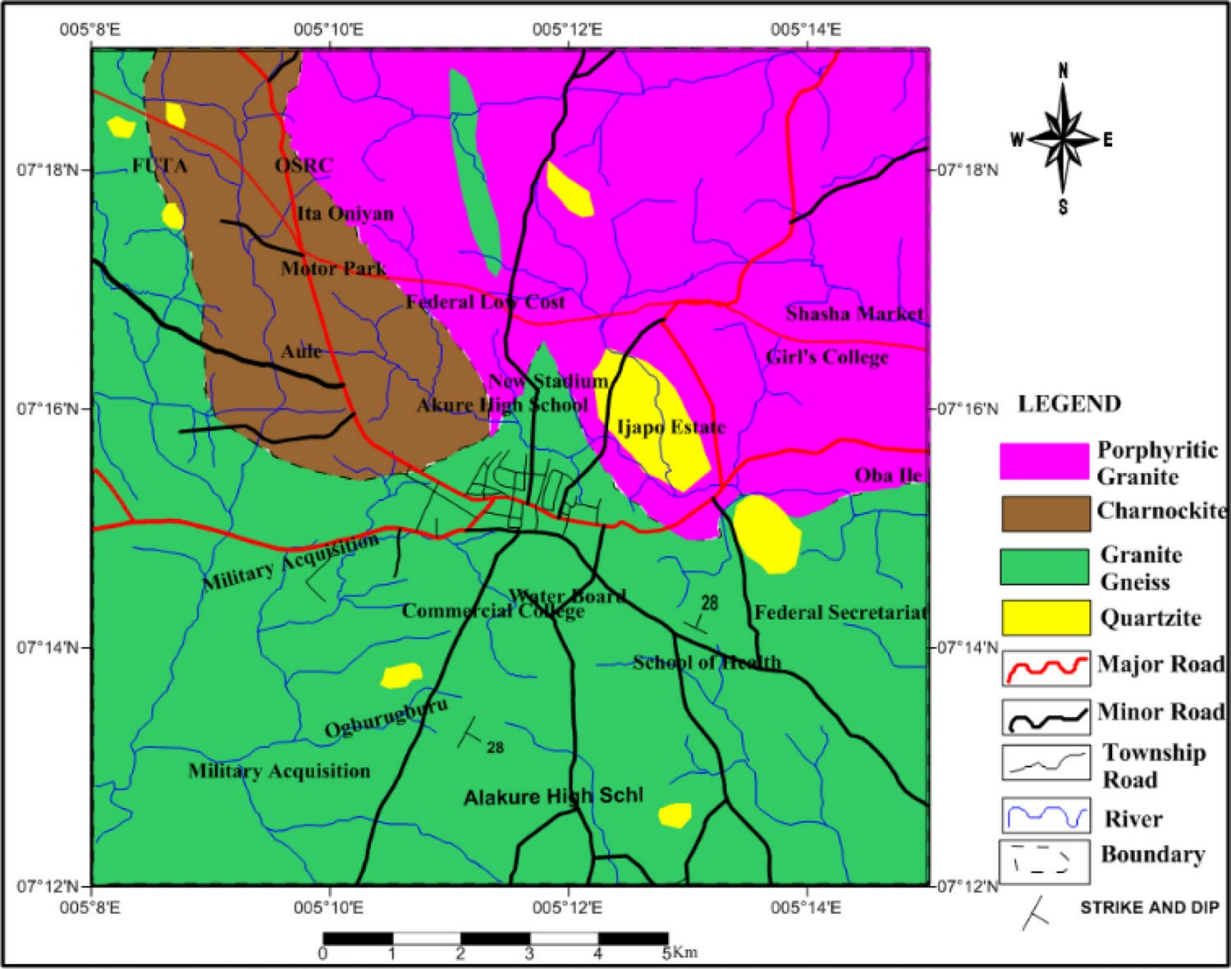
Geological map of the study area^20^**.**

The study area, Akure, is located in Southwestern Nigeria. It lies within latitude 07° 12′ N and 07° 19′ N and longitude 005° 8′ E and 005° 15′ E (Fig. 1). The altitude is about 430 m and the area extent is about 300 square kilometers. The topography is expressed as low-lying relief in the South and Southwest, and an inselberg landscape of high altitude in the North and Northwest^7^. The study area falls within the Precambrian basement complex of Southwestern Nigeria which comprises the Migmatite-gneiss Complex, the Schist Belts, the Older Granites and intruded pegmatites, quartz veins, aplites, and dolerite dykes^18,19^. The predominant rock types underlying the area include; granite gneiss, porphyritic granite, charnockite, and quartzite (Fig. 1). The rock types have been affected by Pan Africa Orogeny (600 Ma), accompanied by regional metamorphism, migmatization, and extensive granitization and gneissification which produced syntectonic granites and homogeneous gneisses^19^. Migmatite-gneiss Complex is the most abundant crystalline rock. The gneisses form an important rock member of the migmatites which differs in terms of their gradation^19^. The porphyritic granite is the most abundant of the older granite suite while medium to coarse-grained granites occur in subordinate amounts. The charnockites are medium to coarse-grained, largely composed of brown to greenish blue quartz and greenish to grey feldspar.

## Methodology

**Figure 2:**
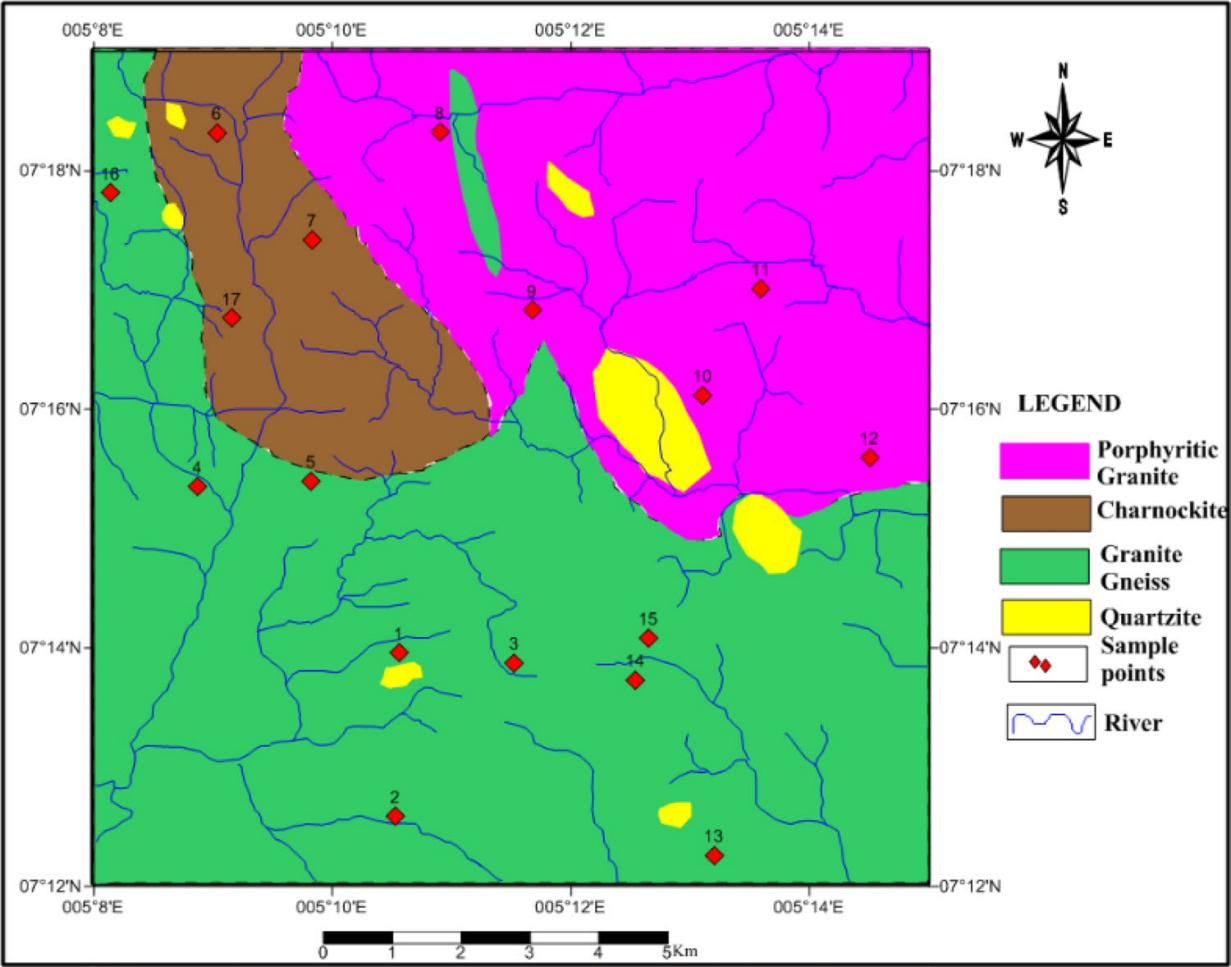
Geological map showing sample locations.

**Figure 3:**
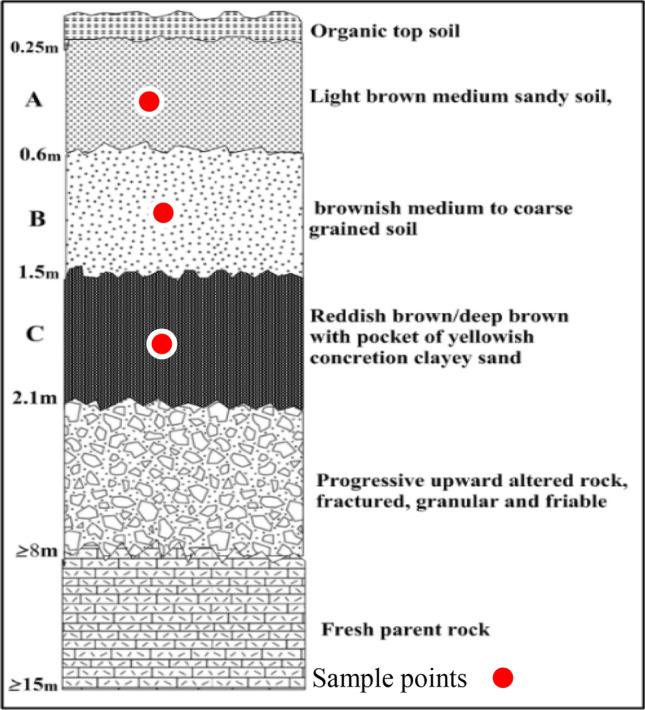
Soil profile developed over granite gneiss (A = Top Accumulation, B = Middle Accumulation and C = Mottled zone).

**Figure 4:**
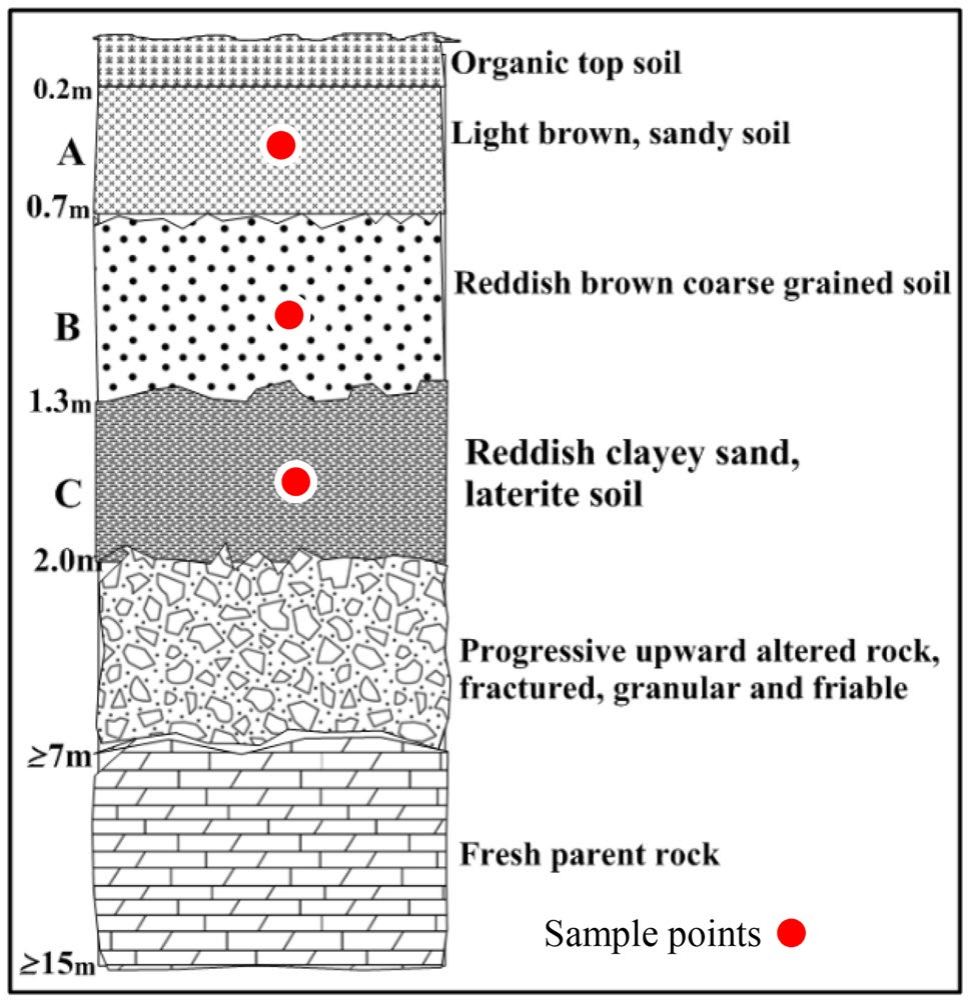
Soil profile developed over porphyritic granite (A = Top Accumulation, B = Middle Accumulation.

**Figure 5:**
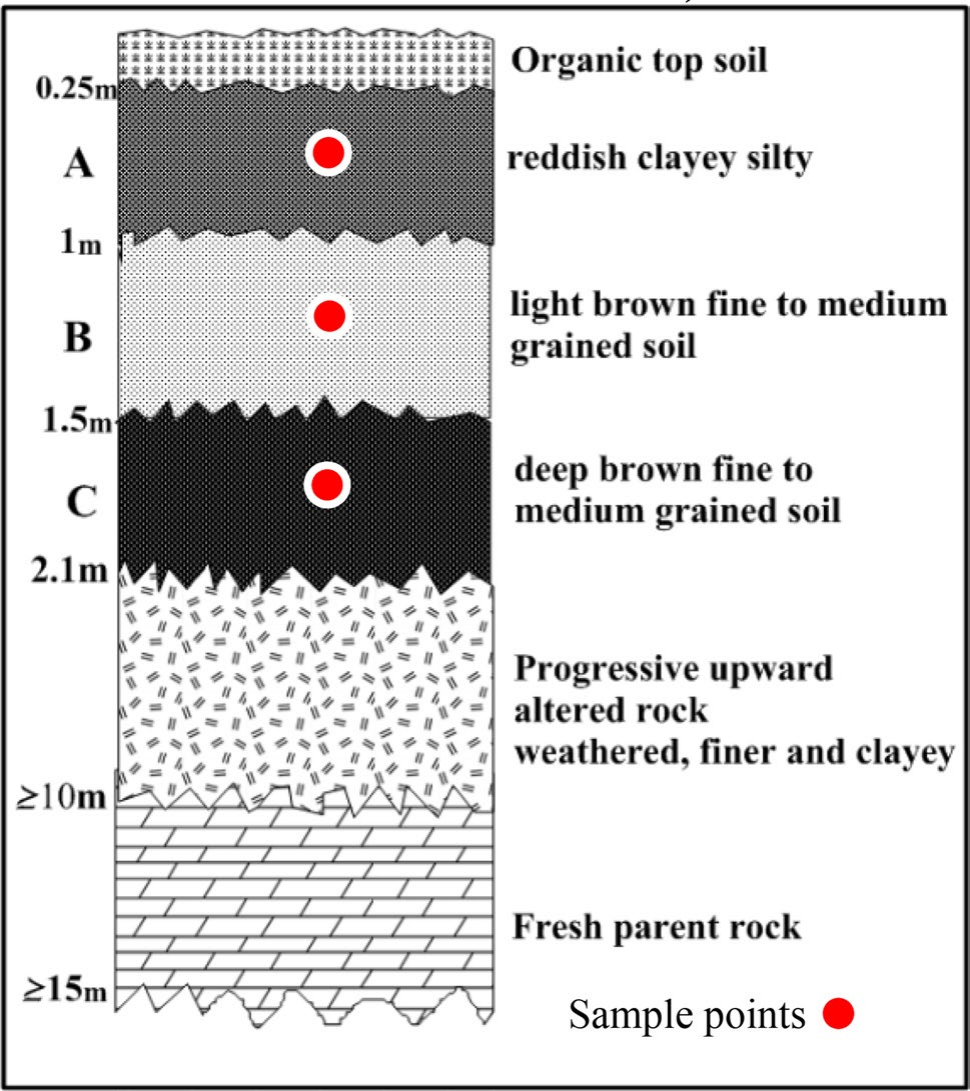
Soil profile developed over charnockite rock (A = Top Accumulation, B = Middle Accumulation and C = Mottled zone).

Fifty-one soil samples were collected from seventeen trial pits at three different depths up to 2 m, see Figs. 2, 3, 4, and 5. These samples were air-dried for two weeks and then transported to the laboratory for analysis. Index engineering properties of soils, including specific gravity, consistency limits (liquid limit, plastic limit, and linear shrinkage), and particle size distribution, were thoroughly investigated. To determine the variation in properties after multiple-compaction, four sets of compaction cycles were carried out using a standard Proctor mold and energy. The sample was removed from the mould and re-compacted with no loss of moisture content after the first component of compaction. After each set of compaction cycles, the soils underwent specific gravity, moisture-density characteristics, particle size distribution, and Atterberg limits tests. The laboratory tests were carried out by BS 13776^21^**.**

### Geo-statistical assessment of soil instability

The effects of mechanical energy on soil properties were analyzed using standard evaluation methods. The evaluation of granulometric modulus and hardness index, based on clay size content, fines content, and coarse content, can accurately assess soil particle disintegration^3,22^. The hardness index (IH) is determined by the ratio of granulometric modulus (Mg) of soil grain sizes before and after applied mechanical energy, indicating the extent of soil particle breakdown by the number of fines produced. Granulometric modulus is expressed as the sum of percentages of materials passing through selected sieve sizes of 4.75 mm, 2 mm, 0.425 mm, and 0.075 mm.

1 $$\left( {{\text{IH}}} \right) = {\text{\% passing }}\frac{{{ }\left( {{\text{size }}(4.75 + 2 + 0.425 + 200} \right){\text{ mm before compaction}}}}{{\left( {{\text{size }}(4.75 + 2 + 0.425 + 200} \right){\text{ mm after compaction}}}}$$

Maximum dry density (MDD), optimum moisture content (OMC), and coefficient of variation (CV) obtained from the moisture-density characteristics have been considered standard methods for evaluating the mechanical instability of soils^23^. The coefficient of variation is expressed in percentage and measured as the standard deviation divided by the mean.

Standard deviation (σ) is given as;

2 $$\frac{1}{{\text{N}}}\mathop \sum \limits_{{{\text{i}} = 1}}^{{\text{N}}} \left( {{\text{X}} - {\overline{\text{X}}}} \right)^{2}$$

where *σ* = standard deviation, X_i_ = each value of the dataset, x̄ = the arithmetic mean of the data, N = the total number of data points, $\mathop \sum \limits_{{{\text{i}} = 1}}^{{\text{N}}} \left( {{\text{X}} - {\overline{\text{X}}}} \right)^{2}$ is the sum of (X-x̄)^2^ for all data points. The mean is given as

3 $${\overline{\text{X}}} = \frac{1}{{\text{N}}}\sum {\text{X}}$$

4 $${\text{Coefficient}}\;{\text{of}}\;{\text{variation}} = \frac{standard\;deviation}{{mean }}{ } \times 100{ }$$

To further standardize these methods, it is important to evaluate the methods with a large number of soil samples derived from different rock types and soil profiles.

## Results and discussions

### Petrography of the parent rocks

**Figure 6:**
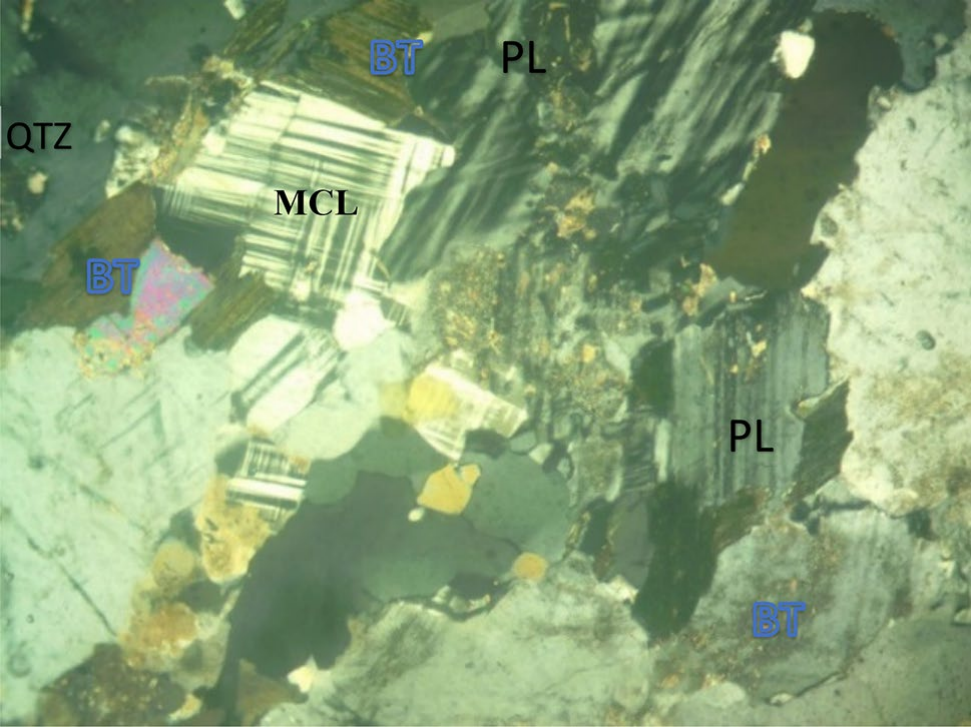
Microphotograph of porphyritic granite (QTZ = quartz, MCL = Microcline, PL = Plagioclase and BT = Biotite).

**Figure 7:**
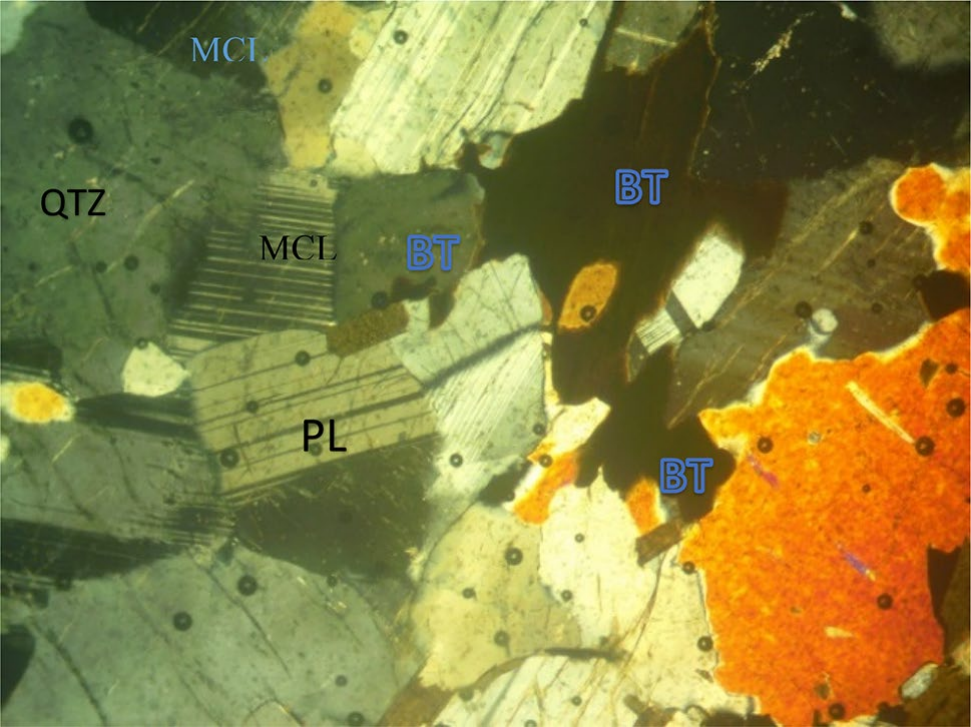
Microphotograph of granite gneiss (QTZ = quartz, PL = Plagioclase, MCL = Microcline and BT = B = Biotite).

**Figure 8:**
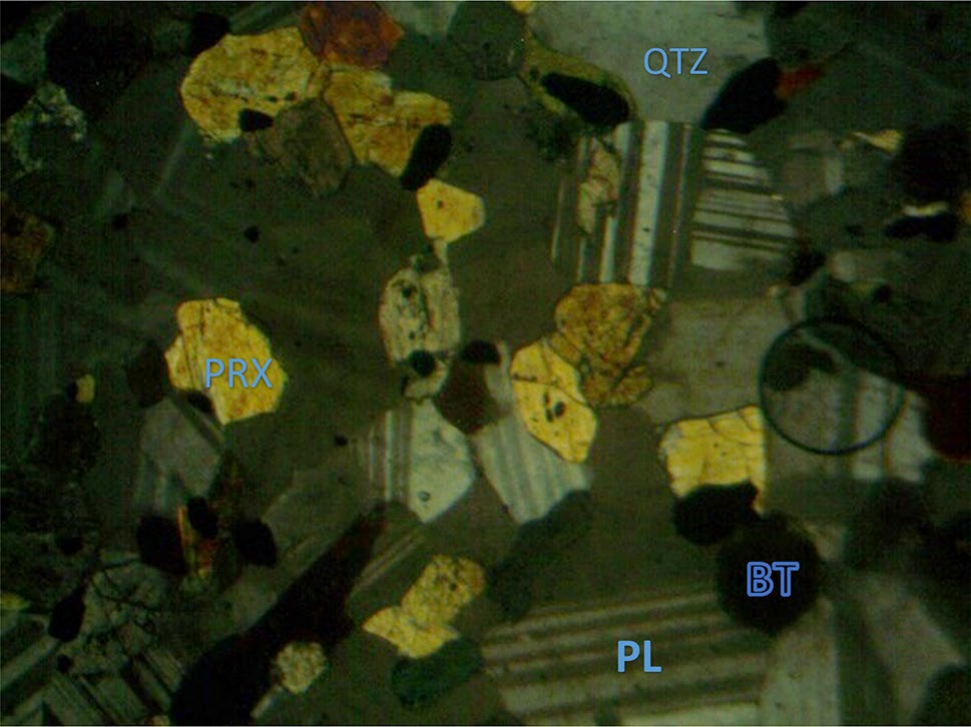
Microphotograph of charnockite (QTZ = quartz, PRX = Pyroxene, PL = Plagioclase and BT = B = Biotite).

**Figure 9:**
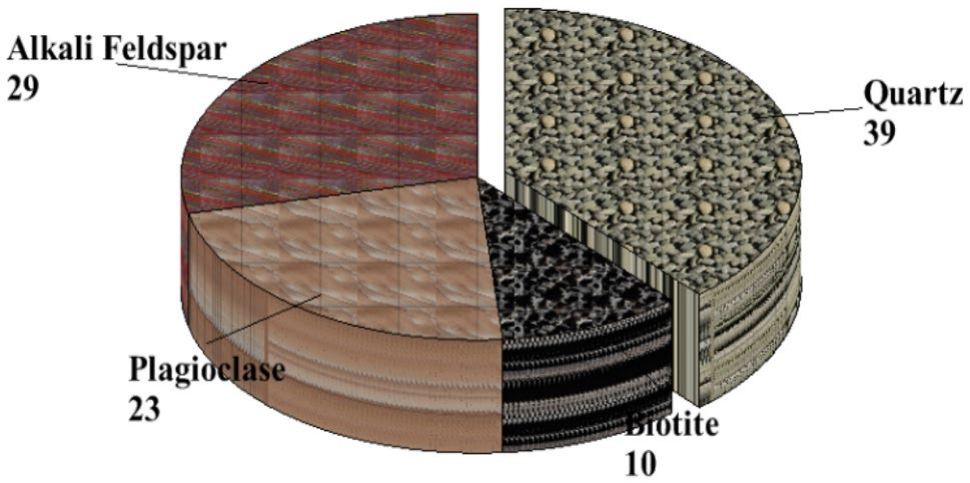
Modal composition for porphyritic granite.

**Figure 10:**
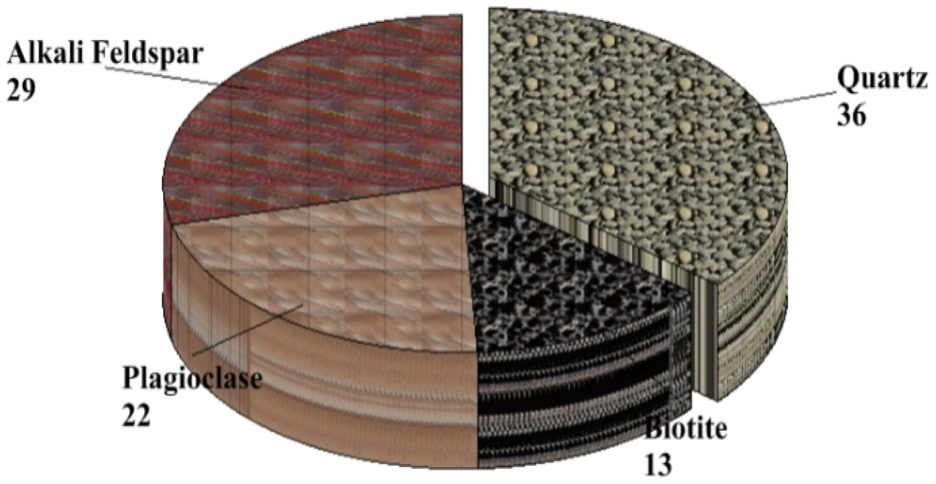
Modal composition for granite gneiss.

**Figure 11:**
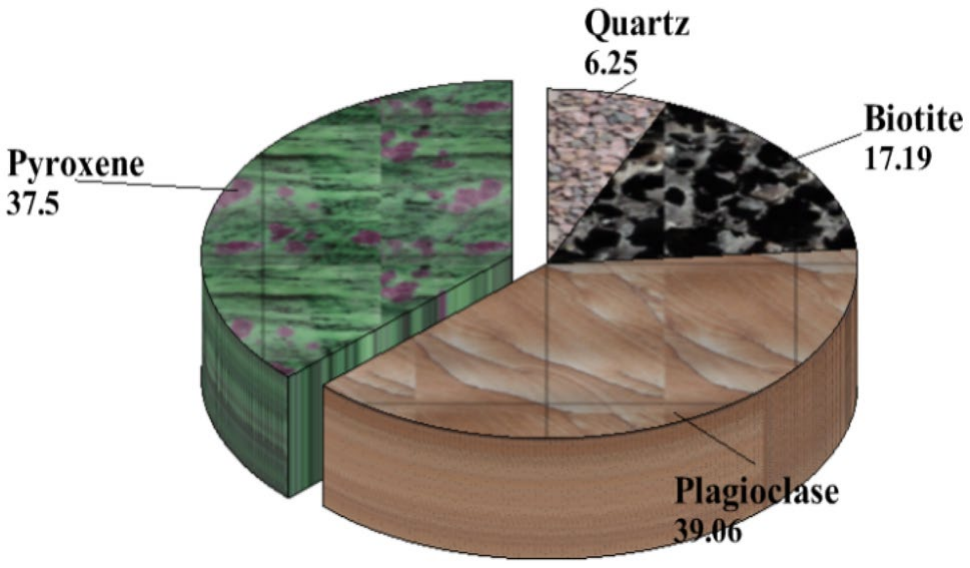
Modal composition for charnockite.

The soil samples are insitu weathered products from granite gneiss, porphyritic granite, and charnockite rocks. The minerals in porphyritic granite are quartz, alkali feldspar, plagioclase feldspar, and biotite (Figs. 6 and 9). The weathered product is composed of medium to coarse grain soils. Granite gneiss may experience a high degree of weathering due to the presence of structural features such as foliations, joints, faults, and folds. Minerals in granite gneiss are quartz, alkali feldspar, plagioclase feldspar and biotite (Figs. 7 and 10). Minerals in charnockite are pyroxene, feldspar, quartz and biotite (Figs. 8 and 11). The weathered products were soils of clayey to fine medium grains. The abundance of feldspars and pyroxene present in charnockite will weather to form clayey soils. The differences in the properties of soils can be attributed to the mineralogy and textural differences in the rock types. The major differences in the clay mineralogy and index engineering properties of the soils can be explained by the differences in the lithology and weathered product of the parent rocks. This implies that structural characteristics, degree of weathering of parent rocks, and mineral composition could influence the stability of soils when modified and compacted (Figs. 9, 10 and 11).

### The specific gravity of the grains

The specific gravity of uncompacted soils ranged from 2.60 to 2.73 while it ranged from 2.63 to 2.75 after the fourth compaction. The values for the uncompacted and compacted soils fall within the reported range of laterite soils of 2.6–2.8^24^. These results show that there is no significant effect of multiple-compaction on the specific gravity despite the disintegration and reduction in porosity of the soils. Das^25^ stated that the specific gravity of quartz minerals is 2.65 while those of montmorillonite and illite fall within 2.62–2.8. The specific gravities for porphyritic granite and granite gneiss-derived soils are typical of the specific gravity of quartz while that of charnockite-derived soils is typical of montmorillonite and illite clay minerals. This explanation may account for the variation in specific gravity of charnockite-derived as compared with those of porphyritic granite and granite gneiss-derived soils.

### Liquid limit

**Figure 12:**
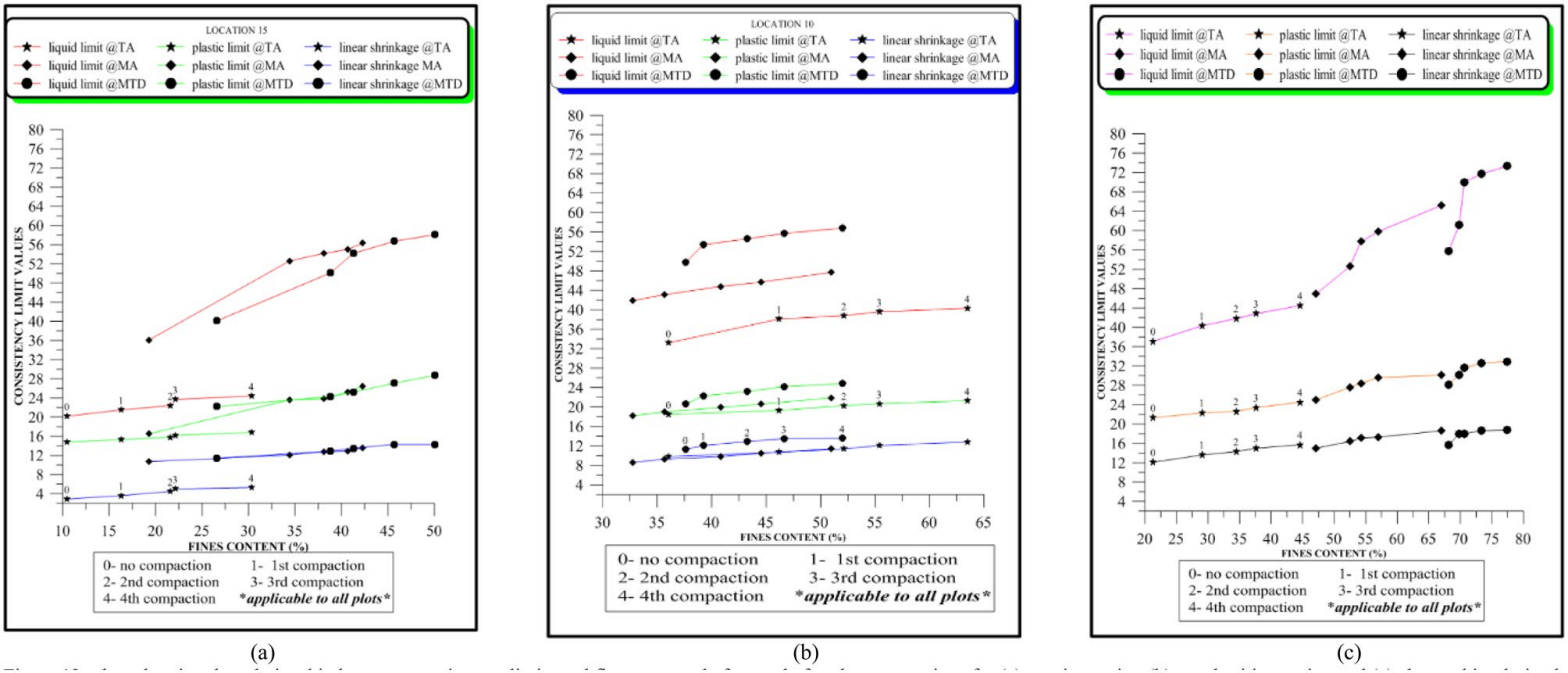
Plots showing the relationship between consistency limits and fines content before and after the compactions for (**a**) granite gneiss, (**b**) porphyritic granite, and (**c**) charnockite-derived soils.

Multiple-compaction produced an increase in the liquid limit values for the soils sampled at all the zones. Liquid limit at the top accumulation zone varied from 18 to 39% before compaction while it varied from 23 to 48% after the fourth compaction (Table 1). Liquid limit at the middle accumulation zone varied from 20 to 47% before compaction, and 28% to 65% after the fourth compaction (Table 2). Liquid limit at the mottled zone varied from 24 to 56% before compaction, and 32% to 73% after the fourth compaction (Table 3). Based on rock type, the liquid limit is higher in charnockite-derived soils as compared to those of porphyritic granite and granite gneiss-derived soils (Fig. 12). The liquid limit is higher in the soils sampled at the mottled zone as compared to the other horizons. The results indicate that multiple increased the percentage of fines. Therefore, an increase in the percentage of fines accounts for a slight increase in the liquid limit. This is consistent with the study of Omotoso^26^ stated that an increase in the amount of fines in the soils will produce a high liquid limit.

**Table 1:**
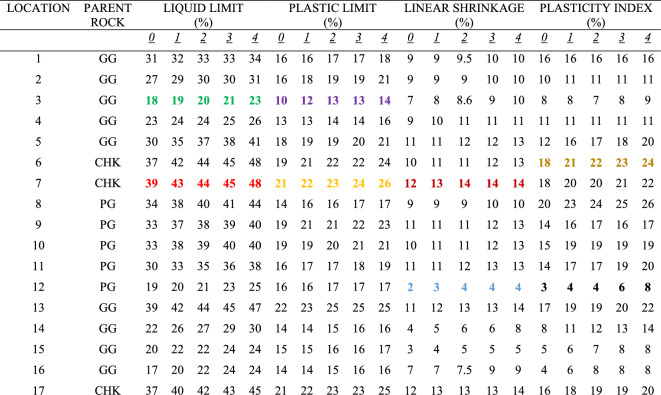
Effect of multiple compaction on consistency limits for the top accumulation zone (TA).

*0 = Without Compaction: 1 = first compaction: 2 = second compaction: 3 = third compaction: 4 = fourth compaction.

(PG = Porphyritic Granite, GG = Granite Gneiss, CHK = Charnockite: The samples that show strong changes with compaction are highlighted in colours: green and red denotes lowest and highest liquid limit; purple and orange denotes lowest and highest plastic limit; blue and brown denotes lowest and highest linear shrinkage; black and dark yellow denotes lowest and highest plasticity index).

**Table 2:**
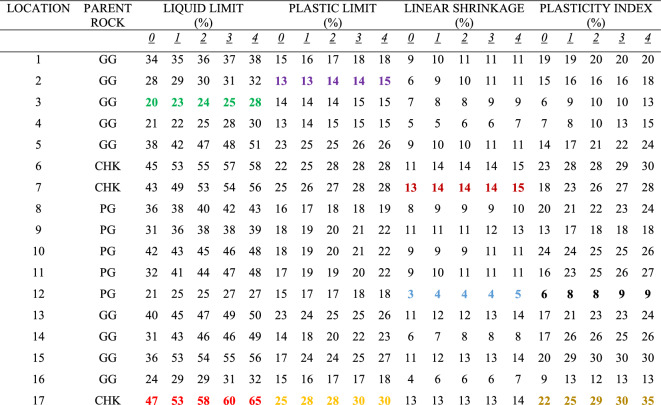
Effect of multiple compaction on consistency limits for the middle accumulation zone (MA).

*0 = Without Compaction: 1 = first compaction: 2 = second compaction: 3 = third compaction: 4 = fourth compaction.

(PG = Porphyritic Granite, GG = Granite Gneiss, CHK = Charnockite: The samples that show strong changes with compaction are highlighted in colours: green and red denotes lowest and highest liquid limit; purple and orange denotes lowest and highest plastic limit; blue and brown denotes lowest and highest linear shrinkage; black and dark yellow denotes lowest and highest plasticity index).

**Table 3:**
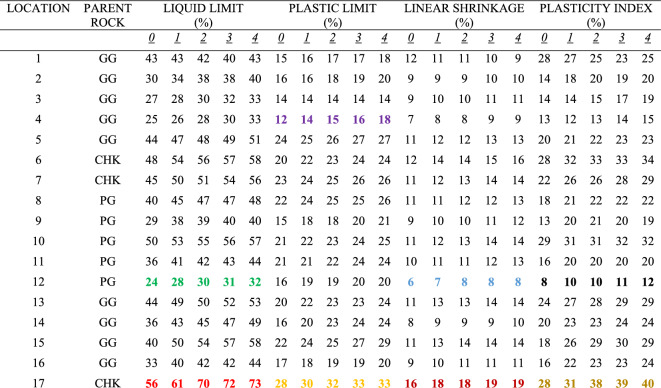
Effect of multiple compaction on consistency limits for the mottled zone (MTD).

*0 = Without Compaction: 1 = first compaction: 2 = second compaction: 3 = third compaction: 4 = fourth compaction.

(PG = Porphyritic Granite, GG = Granite Gneiss, CHK = Charnockite: The samples that show strong changes with compaction are highlighted in colours: green and red denotes lowest and highest liquid limit; purple and orange denotes lowest and highest plastic limit; blue and brown denotes lowest and highest linear shrinkage; black and dark yellow denotes lowest and highest plasticity index).

### Plasticity index and linear shrinkage

Multiple-compaction produced an increase in the plasticity index for the soils sampled at all the zones. The plasticity index at the top accumulation zone varied from 3 to 8% before compaction, and 18% to 24% after the fourth compaction. The plasticity index at the middle accumulation zone varied from 6 to 22% before compaction, and 9% to 35% after the fourth compaction. For the mottled zone, it varied from 8 to 28% before compaction and, 12% to 40% after the fourth compaction. Charnockite-derived soils have a higher plasticity index as compared to those of granite gneiss and porphyritic granite-derived soils. The linear shrinkage changes after multiple-compaction for all the zones sampled. For the top accumulation zone, it varies from 2 to 12% before compaction and 4% to 14% after the fourth compaction. At the middle accumulation zone, linear shrinkage varied from 3 to 13% before compaction and 5% to 15% after the fourth compaction. Linear shrinkage at the mottled zone varied from 6 to 16% before compaction and 8% to 19% after the fourth compaction. Soils developed over porphyritic granite have the lowest linear shrinkage as compared to those of granite gneiss and charnockite-derived soils (Fig. 12). Linear shrinkage is higher in the mottled zone as compared to the other sampled horizons. An increase in the linear shrinkage of the soils corresponds to an increase in the plasticity index. The results for the plasticity index and the linear shrinkage are presented in Tables 1, 2, 3

### Particle size distribution

**Figure 13:**
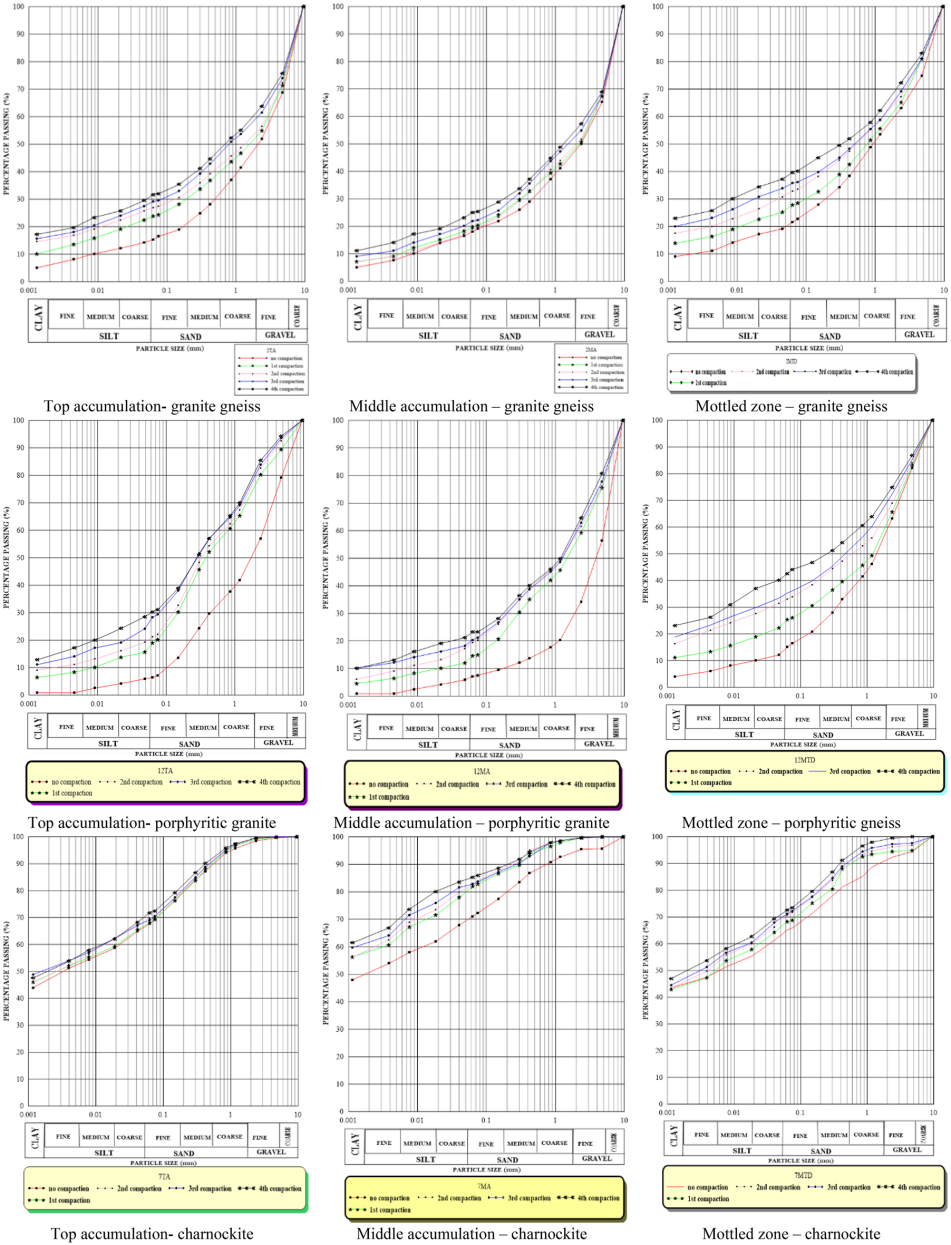
Particle size distribution for the derived soils before and after the compactions. (CPT 1 = First compaction cycle; CPT 2 = Second compaction cycle; CPT 3 = Third compaction cycle and CPT 4 = Fourth compaction cycle).

Multiple-compaction on soil samples shows that there is a breakdown of the coarse content to fine particles and a further breakdown of the fines. Coarse content at the top accumulation zone varied from 1.5% to 56.1% before compaction, and 0.5% to 24.3% after the fourth compaction. The fines content varied from 6.4% to 67.6% before compaction; and 30.3% to 71.8% after the fourth compaction. For the middle accumulation zone, the coarse content varied from 4.6% to 65.3% before compaction; and 0.3% to 35.4% after the fourth compaction. The fines content varied from 7.1% to 71.0% before compaction; and 23.2% to 85.3% after the fourth compaction. Coarse content at the mottled zone varied from 1.12% to 41.2% before compaction and, 0.4% to 33.2% after the fourth compaction. The fines content varied from 26.8% to 69.8% before compaction, and 38.2 to 77.4% after the fourth compaction. The particle size distribution curves (Fig. 13) for all the horizons before and after the compactions indicate the responses of the particles to compactions. The results show that multiple-compaction increases the amount of fines across the three soil types. The weakly-bound micro-clusters in the charnockite soils are disintegrated into finer particles when compacted. The influence of multiple-compaction on particle sizes was assessed by the Hardness Index (IH). The hardness index is low in porphyritic granite and granite gneiss-derived soils as compared to those of charnockite-derived soils. This indicates that soils of fine grains tend to produce a higher hardness index than coarse-grain soils (Table 4). Malomo^3^ stated that the implication of high hardness index values (above 0.8) is that soil particles become better graded with the increase in the number of compaction. Therefore, multiple-compaction has a significant effect on the particle size distribution.

**Table 4:** Percentage of fines and hardness index based on the rock type.

Location	Lithology	Percentage of fines			Hardness index		
		TA	MA	MTD	TA	MA	MTD
Percentage of fines and hardness index of soils derived from granite gneiss							
1	Granite gneiss	46	44	48	0.973	0.89	0.83
2	Granite gneiss	26	21	33	0.827	0.92	0.86
3	Granite gneiss	37	35	39	0.957	0.98	0.97
4	Granite gneiss	29	29	31	0.861	0.897	0.92
5	Granite Gneiss	53	34	35	0.882	0.872	0.78
13	Granite Gneiss	59	38	34	0.912	0.897	0.84
14	Granite Gneiss	35	24	34	0.864	0.853	0.91
15	Granite Gneiss	20	35	40	0.668	0.739	0.76
16	Granite Gneiss	55	42	49	0.956	0.965	0.98
Percentage of fines and hardness index of soils derived from granite gneiss							
8	Porphyritic Granite	51	36	33	0.904	0.942	0.9
9	Porphyritic Granite	44	38	40	0.901	0.911	0.92
10	Porphyritic Granite	51	41	43	0.907	0.939	0.94
11	Porphyritic Granite	43	37	42	0.883	0.931	0.94
12	Porphyritic Granite	21	17	30	0.676	0.568	0.82
Percentage of fines and hardness index of soils derived from granite gneiss							
6	Charnockite	32	50	38	0.894	0.927	0.94
7	Charnockite	69	80	70	0.988	0.928	0.94
17	Charnockite	33	55	71	0.811	0.87	0.98

*TA = Top Accumulation, MA = Middle Accumulation, MTD = Mottled Zone.

### Compaction characteristics

**Figure 14:**
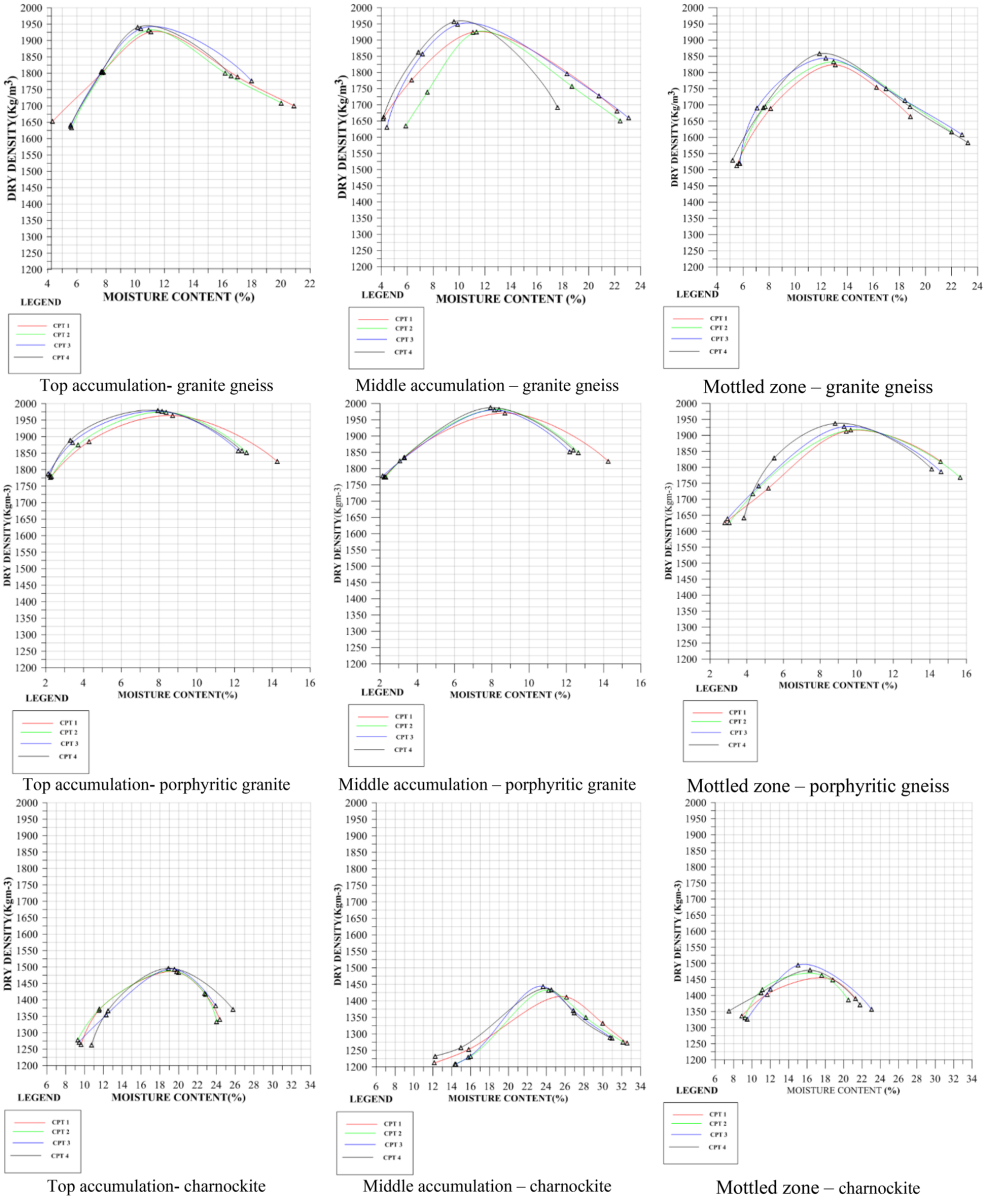
Effect of multiple-compaction based on density-moisture relationship.

The average maximum dry density in granite gneiss-derived soil varied across different zones: 1705.3 to 2053.5 for the top zone, 1760.8 to 1966.5 for the middle accumulation zone, and 1652.5 to 1908.8 for the mottled zone. For porphyritic granite-derived soils, the average maximum dry density ranged from 1656.8 to 1974 for the top zone, 1725.3 to 1980.5 for the middle accumulation zone, and 1704 to 1922 for the mottled zone. Lastly, in charnockite-derived soil, the average maximum dry density ranged from 1490 to 1784.3 for the top zone, 1431.5 to 1647 for the middle accumulation zone, and 1436 to 1614.5 for the mottled zone. Figure 14 shows the moisture-density curves for granite gneiss, charnockite, and porphyritic granite-derived soils. The maximum dry density of the soil increases with the compaction cycle, while the moisture content slightly decreases. The maximum dry density of granite gneiss and porphyritic granite-derived soils is higher than that of charnockite-derived soil. This discrepancy is due to the mineral composition in the parent rock. Charnockite, which contains abundant pyroxene and plagioclase feldspar, disintegrates more easily during weathering, resulting in a cluster of clayey materials that can further break down with increased energy levels.

Table 5 presents the effect of multiple compaction on the soils using the coefficient of variation (CV). For granite gneiss-derived soils, the coefficient of variation for maximum dry density ranged from 0.28% to 1.09%, 0.09% to 1.86% and 0.36% to 0.86% for the top, middle accumulation and mottled zones, respectively. The CV for optimum moisture content varied from 1.73% to 6.71%, 1.13% to 10.4% and 1.44% to 7.45% for the top, middle and mottled accumulation zones, respectively. In porphyritic granite-derived soils, the CV for maximum dry density ranged from 0.31% to 1.58%, 0.28% to 2.13% and 0.41% to 0.86% for the top, middle and mottled accumulation zones, respectively. For optimum moisture content, CV ranged from 3.55% to 10.3%, 2.47% to 10.4% and 0.51% to 9.48% for the top, middle and mottled accumulation zones, respectively. In charnockite-derived soils, the CV for maximum dry density varied from 0.31% to 0.98%, 0.51% to 0.96% and 0.26% to 1.13% for the top, middle and mottled accumulation zones, respectively. The CV values for optimum moisture content ranged from 0.46% to 9.1%, 0.26% to 3.4% and 0.6% to 5.4% for the top, middle, and mottled accumulation zones, respectively.

**Table 5:** Coefficient of variation for repeated compaction based on the rock type.

Rock Type	Maximum Dry Density (MDD) (Kg/m^3^)			Optimum Moisture Content (OMC) (%)			Coefficient of Variation for the MDD (%)			Coefficient of Variation for the OMC (%)		
	TA	MA	MTD	TA	MA	MTD	TA	MA	MTD	TA	MA	MTD
Granite gneiss	1705.3	1760.8	1699.5	15.1	13	14.48	0.291	1.56	0.36	5.38	4.22	4.38
Granite gneiss	1933.5	1940	1839.8	10.8	11	12.55	0.28	0.79	0.67	2.85	6.98	3.63
Granite gneiss	1830.7	1855.8	1791.5	11.3	11	12.75	0.836	1.06	0.86	6.71	5.95	4.38
Granite gneiss	1882.8	1933.8	1833.8	12.2	9.6	12.75	0.684	0.16	0.85	5.71	3.81	6.5
Granite gneiss	1798.3	1802.3	1810	14.7	14	14.3	0.763	0.56	0.7	2.59	1.83	6.14
Granite gneiss	1820	1849	1795.5	14	11	14.7	0.551	1.86	0.47	6.11	10.4	3.12
Granite gneiss	2053.5	1966.5	1908.8	9.25	11	11.2	0.615	0.48	0.68	6.19	3.97	5.61
Granite gneiss	1914.3	1901.8	1652.5	10.8	13	16	0.292	0.84	0.83	1.73	5.77	7.45
Granite gneiss	1787	1909.8	1734.5	11.7	9.6	14.18	1.087	0.09	0.37	5.29	1.13	1.44
Porphyritic granite	1857.5	1874	1817.8	10.2	10	10.13	0.411	1.18	0.43	10.3	10.4	9.48
Porphyritic granite	1760.3	1752.8	1808.5	15.8	15	13.25	1.578	0.82	0.45	3.55	5.33	1.89
Porphyritic granite	1711.8	1737.8	1704	15.1	14	17.43	1.234	2.13	0.41	6.66	12.1	2.51
Porphyritic granite	1656.8	1725.3	1708.8	17.5	15	14.35	0.918	0.38	0.86	4.52	2.47	6.98
Porphyritic granite	1974	1980.5	1922	8.23	8.2	8.93	0.306	0.28	0.5	4.58	5.79	4.91
Charnockite	1784.3	1647.8	1614.5	13.4	14	18.13	0.883	0.51	0.79	9.1	2.59	4.93
Charnockite	1490	1431.5	1471.8	19.1	24	16.63	0.309	0.81	1.13	0.46	3.43	5.37
Charnockite	1576.3	1549	1436	17.6	19	24.33	0.975	0.96	0.26	4.2	2.9	0.61

TA = Top Accumulation, MA = Middle Accumulation, MTD = Mottled Zone and LC = Locations: GG = Granite Gneiss, PG = Porphyritic Granite, CHK = Charnockite.

The coefficient of variation for the maximum dry density and optimum moisture content of soils developed over granite gneiss is higher in the middle accumulation zone compared to soils obtained from the top and mottled accumulation zones. The coefficient of variation for the maximum dry density and optimum moisture content of soils developed over porphyritic granite is higher in the middle accumulation zone compared to the top and mottled accumulation zones. The coefficient of variation for the maximum dry density of soils developed over charnockite is higher in the mottled accumulation zone compared to the soils obtained from the top and middle accumulation zones. For optimum moisture content, the variation is high in the top accumulation zone compared to the middle and mottled zones.

According to Eze et al.^23^, a coefficient of variation of at least 10% is considered a significant level of influence for statistical analysis. Therefore, the results obtained from the coefficient of variation indicate that multiple-compaction did not significantly affect the maximum dry density and optimum moisture content, despite the disintegration of particle sizes. Based on the study of Li et al.^8^, the suitable maximum dry density (MDD) and optimum moisture content (OMC) when using standard proctor test methods are classified based on the material type. For clay, the maximum dry density falls between 1440 kg/m^3^ and 1685 kg/m^3^, and the optimum moisture content may range between 20 and 30%. For silty clay, the maximum dry density is between 1600 kg/m^3^ and 1845 kg/m^3^, and the optimum moisture content ranges between 15 and 25%. For sandy clay, the maximum dry density ranges between 1750 kg/m^3^ and 2165 kg/m^3^, and the optimum moisture content is between 8 and 15%. From the moisture-density relationship, it can be deduced that soils derived from porphyritic granite and granite gneiss are silty clay or sandy clay, while soils derived from charnockite are clayey soils.

### Clay minerals and activity

**Figure 15:**
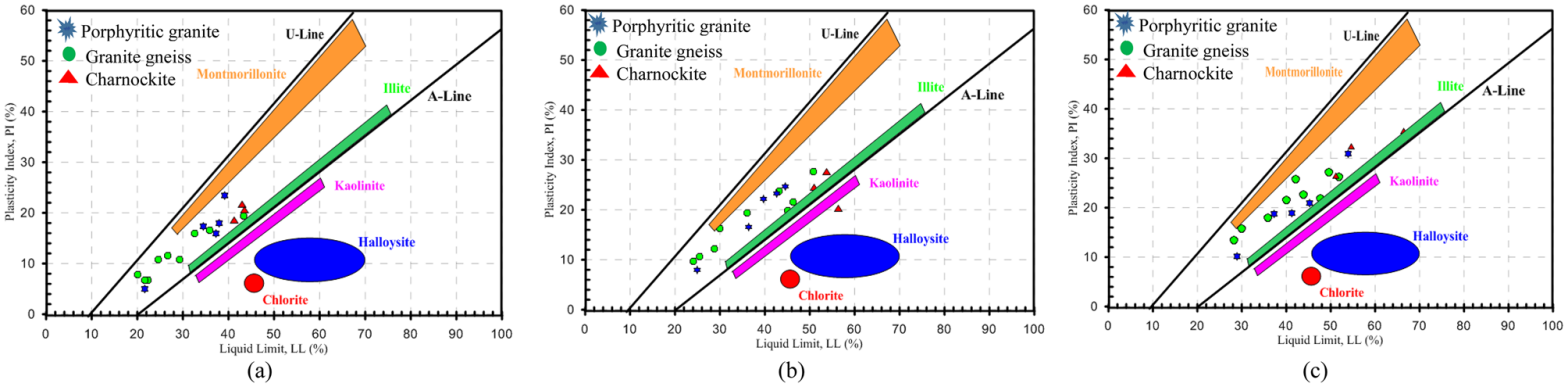
Clay minerals identification method using plasticity chart: (**a**) Top accumulation, (**b**) Middle accumulation and (**c**) Mottled zone.

**Figure 16:**
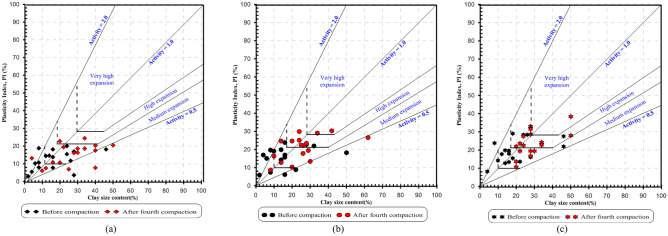
Effect of compaction on clay activity (**a**) Top accumulation, (**b**) Middle accumulation and (**c**) Mottled zone.

The graphical representation of the plasticity index and liquid limit demonstrates the accurate identification of clay minerals namely, Montmorillonite, illite, and kaolinite (Fig. 15). It is a fast and cost-effective method of identifying the mineral phases in the soil. Montmorillonite and illite minerals typically display significant shrinkage and swelling properties due to the replacement of silica and alumina ions, high water levels, and an excess of negative charge. Kaolinite has less cation exchange capacity (CEC), low surface area and low isomorphous substitution, and low plasticity, cohesion, shrinkage, and swelling characteristics^27^. The clay's activity is a clear indication of the swelling potential of soils. It has been observed that uncompacted soils exhibit medium to high expansion potential (activity between 0.5 and 1.0). However, after the fourth compaction, the soils become highly active with a very high expansion potential (activity between 1 and 2) (Fig. 16). Therefore, multiple compactions lead to a significant increase in soil activity. The activity chart that shows the effect of multiple compaction on plasticity index and clay size content for all the horizons is presented in Fig. 16.

## Conclusion

Through multiple-compaction, larger grains in soils derived from granite gneiss and porphyritic granite broke down into smaller particles, which were evident by the granulometric modulus and hardness index. However, in charnockite soils, the fine grains that are not clustered broke down more easily than the large grains of quartz that are bound together by clayey materials. The differences in consistency limits and particle size distribution characteristics of soils are directly related to the parent rock from which they were derived. Interestingly, despite multiple rounds of compaction, the maximum dry density (MDD) and optimum moisture content (OMC) remained consistent. The fine grains filled up the available pore spaces in the sample, allowing the compaction characteristics to remain consistent with an increase in the energy level. The coefficient of variation of the compaction results indicated that an increase in the number of compactions did not significantly affect the maximum dry density and optimum moisture content of the soils. For highway construction materials, the middle accumulation zone is preferred because it has a higher maximum dry density, coarse content and lower optimum moisture content. Pedogenesis of the parent rock has caused differences in the consistency limits and particle size distribution characteristics of soils developed from porphyritic granite, granite gneiss, and charnockite. Based on the densification and the stable behaviour of the derived soils from different parent rocks under multiple-compaction, the research shows that porphyritic granite and granite gneiss-derived soils are more suitable as engineering materials than soils derived from charnockite.

## Data Availability

The datasets used and/or analyzed during the current study are available from the corresponding author upon reasonable request.
